# Toledo Dental Society

**Published:** 1897-01

**Authors:** 


### Toledo Dental Society.

The Toledo Dental Society held its regular monthly meeting on Friday afternoon and evening, December 11, 1896. The afternoon meeting was devoted to clinics. A very interesting and instructive clinic was given by Dr. C. H. Harroun, in which quite a difficult operation was performed in a pulpless, first superior bicuspid. The method of treatment and principles involved were very lucidly explained by the Doctor, and the operation was an eminent success. The Doctor's method of introducing the filling was different from that usually employed.

He uses a combination of hand-force with the mallet. His filling was composed of gold and tin, and more than ordinary skill was displayed in the operation. Some of the filling instruments employed by the Doctor were of unusual form, and seemed well adapted for the manner of his manipulation. All who witnessed the operation expressed themselves as greatly gratified, and every one who saw it was both instructed and stimulated.

The construction and adaptation of a porcelain crown to the root of the central superior incisor was accomplished by Dr. Allan, of Toledo. By this process the largest practicable amount of the crown of the natural tooth is preserved. The palatine portion of the root is permitted to remain and made to serve as a strong support for the retention of the crown. A band, closely fitted, was attached to the palatine portion which remained and came down the sides well-nigh to the front. The end of the stump was dressed obliquely to the axis of the tooth by a plain, smooth surface from the anterior margin of the gum to the opposite side, leaving the inner or palatine portion standing about half way from the neck of the tooth to its cutting edge. This beveled portion was covered with a platinum plate, about No. 30, burnished down to the edge of the band and soldered to it. The post or pin passed through this plate into the root and soldered to the plate. A porcelain tooth (a common, plain, plate-tooth of proper size, shape and color), was fitted to its right position, then backed and soldered, the palatine part being filled with soldier to give the shape of the natural tooth. The crown thus made, when cemented to the root, presented an entirely natural appearance and was very firmly attached. Dr. Allan received the hearty thanks of every member for this operation.

The evening session was also a very interesting one, a paper being read on "Antiseptics and Disinfectants," upon which a very interesting discussion arose, in which most of the members took part. Every member present felt that he had been well repaid for the expenditure of time and money involved by his attendance. One very encouraging feature of this Society is that a part of its membership is composed of dentists who come from surrounding towns, some twenty or thirty miles distant. Another encouraging feature is that all the members seem determined to make this Society an eminent success, and it will be done. This is only an illustration of what could, and ought to be, done by every town or city in the State having forty thousand inhabitants or more—and, indeed, by smaller places, and we would suggest to all such to make an effort in this direction. If an earnest effort were made, the success that would be attained would be a matter of surprise even to those who are engaged in it. This is merely a suggestion, but we hope it may be acted upon in every town where it would seem feasible. Wherever ten or fifteen reputable dentists could be gathered there should be a society, and this should be borne in mind: That those who are interested in this kind of work are usually ready to respond to any calls for assistance in such work.

